# Identifying miRNA/mRNA negative regulation pairs in colorectal cancer

**DOI:** 10.1038/srep12995

**Published:** 2015-08-13

**Authors:** Xile Zhou, Xiangming Xu, Jinhai Wang, Jianjiang Lin, Wenbin Chen

**Affiliations:** 1 Department of Colorectal Surgery, the First Affiliated Hospital, College of Medicine, Zhejiang University, 79 Qingchun Road, Hangzhou, Zhejiang 310003, P.R. China

## Abstract

Although considerable progress has been made in the molecular biology of Colorectal cancer (CRC), novel approaches are still required to uncover the detailed molecular mechanism of CRC. We aim to explore the potential negatively regulated miRNA-mRNA pairs and investigate their regulatory roles so as to elaborate the potential roles of the critical proteins in the signaling pathways enriched by the differential target genes of negatively regulated miRNA in CRC. Firstly, the differential miRNA-mRNA pairs were selected, followed by pairs of miRNA and their target genes. The obtained relationships were subjected to do functional enrichment analysis and those enriched in CRC pathways were chose to further construct a protein interaction network. Finally, we analyzed the regulatory roles of these relationships and constructed a regulatory network of negatively regulated miRNA and mRNA relationships. A total of 372 pairs of miRNA-mRNA were found and 108 target genes of miRNA were obtained. Three miRNAs including hsa-mir-23b, hsa-mir-365-1 and hsa-mir-365-2 showed significant influence on prognosis of CRC patients. To conclude, the miRNA/mRNA deregulations pairs identified in this study have high potentials to be further applied in diagnosis and treatment of CRC.

Colorectal cancer (CRC) is the third leading cause of cancer-related death worldwide^1^ and recently accumulated evidence indicates that CRC is a genetically heterogeneous and complicated disease caused by abnormalities in gene structure and/or expression^2^. It is now generally accepted that CRC mainly develops through two different genetic pathways, of which one is the chromosomal instability pathway characterized by the involvement of APC, p53, and k-ras genes, by 18q allelic loss, and by aneuploidy DNA content, while the other is a pathway involving microsatellite instability (MSI)^3^. Although considerable progress has been made in molecular biology, novel approaches are still required to uncover the detailed molecular mechanism of CRC.

Recently, microarray tools have been enriched by the development of platforms for the analysis of miRNA expression^4^. miRNAs consists of an abundant class of endogenous, small non-coding RNAs at a length of 18–25 nucleotides, which can repress protein translation by binding to the target mRNAs. It is reported there are over 700 miRNA sequences in the human genome^5^ by the latest version of miRBase (release 13.0, March 2009). In addition, miRNAs have been mainly studied in the field of oncological research, and more and more evidence shows that altered miRNA regulation may involve in the pathogenesis of cancers through regulating the translation of tumor suppressors and oncogenes^6^,^7^,^8^. In addition, miRNAs can target up to several hundred mRNAs, which makes them very powerful regulators and an aberrant miRNA expression can disturb a multitude of cell signaling pathways and profoundly influence cancer onset and progression. Changes in the expression of miRNAs have been observed in a variety of human tumors. Although the expression differences can not represent causal events of carcinogenesis necessarily, yet these changes may regulate some genes that are very important during the process of tumor pathogenesis and may contribute to the classification and prognosis of tumors. These alterations of miRNA expression^7^,^8^ have now been detected in various solid tumors and hematological malignancies, such as CRC. As is known, the main function of mammalian miRNAs is post-transcriptionally regulating their target mRNAs, indicating the combination of mRNAs expression and miRNAs may represent the transcriptional program which describes the normal and tumor tissue characteristics more accurately. At present, there are two approaches applied to study the connection between miRNAs and CRC, including functional and profiling studies. Anyhow, it is showed that the expression profiles of miRNAs have the same potentiality to identify the biomarkers as profiling of their mRNA or protein counterparts. This enables prognosis prediction, therapy response and distinguishing some kinds of disease like CRC.

In this study, we analyzed the expression data of miRNAs and mRNAs of CRC in (The Cancer Genome Atlas) TCGA database so as to excavate the potential deregulated miRNA/mRNA and expound the possible roles of the critic proteins in the signaling pathways enriched by the differential target genes of the deregulated miRNA, hoping to better understand the pathogenesis and make contributes to the diagnosis or therapy of CRC.

## Results

### Differential genes and miRNA

**Figure 1:**
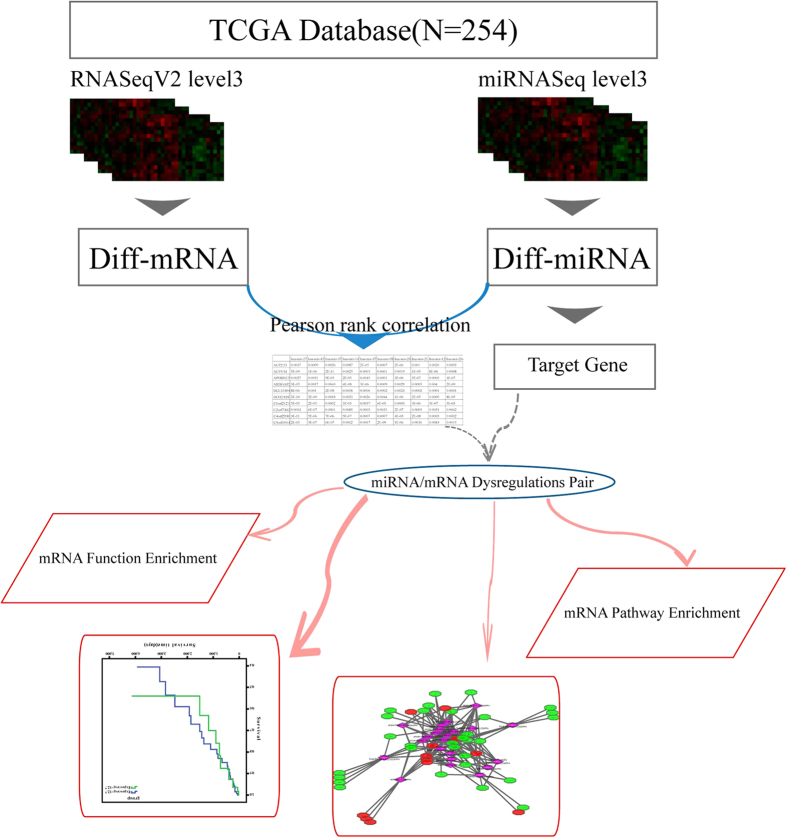
Flowcharts for obtaining the expression data of genes and miRNA.

**Figure 2:**
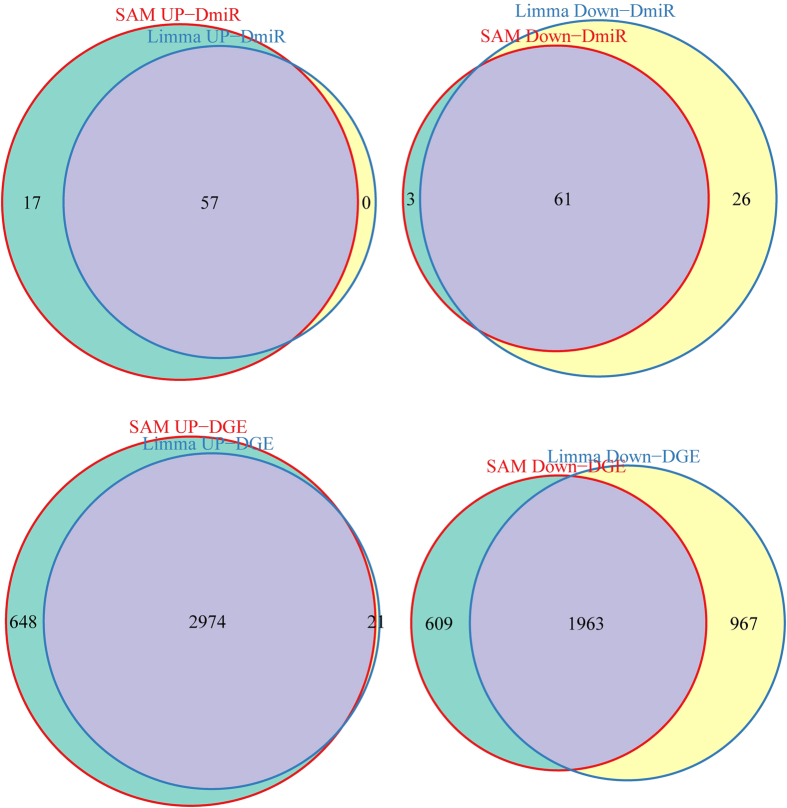
The differential genes and miRNA screened by SAM and limma algorithms. The four crossed regions marked in purple represented the up-regulated differential miRNA, down-regulated differential miRNA, up-regulated differential genes and down-regulated differential genes respectively.

**Figure 3:**
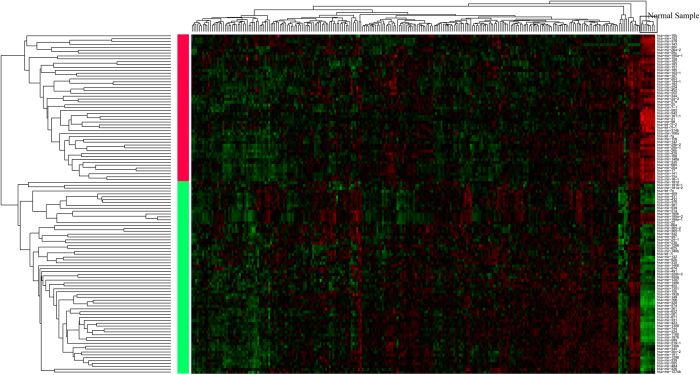
Heat map of microarray results of the differential miRNA. The right longitudinal axis showed the names of miRNA; the left longitudinal axis showed the clustering information of miRNA; the upper abscissa axis showed the clustering information of samples. Red represented the up-regulated miRNA while green represented the down-regulated miRNA. The clustering of samples were mainly divided into two major clusters, one was the normal tissue samples and the other was cancer tissue samples; the clustering of miRNA was the same with that of samples, one was the up-regulated miRNA in cancer tissues while the other was the down-regulated miRNA.

The information of expression value of 20531 genes and 680 miRNAs in a total of 253 samples was obtained after pre-processing the TCGA expression data with the detailed flowcharts shown in Fig. 1. Then the differential genes and miRNAs were screened by SAM and limma algorithms was/were shown in Fig. 2. A total of 4937 differentially expressed genes were selected by both the algorithms, among which 2974 of genes were up-regulated while 1963 of genes were down-regulated in cancer samples, accounting for 60.23% and 39.77% of the total differential genes respectively. There were 118 differentially expressed miRNAs, among which 57 were up-regulated while 61down-regulated in cancer samples, accounting for 48.31% and 51.69% of the total differential miRNAs (Table 1). In order to better characterize the differential miRNAs to distinguish between cancer and normal samples, the differential miRNAs were further used to do clustering analysis. The differential miRNAs could distinguish the normal and cancer samples effectively, as shown in Fig. 3.

### Functional enrichment analysis of the differential targeted genes

**Figure 4:**
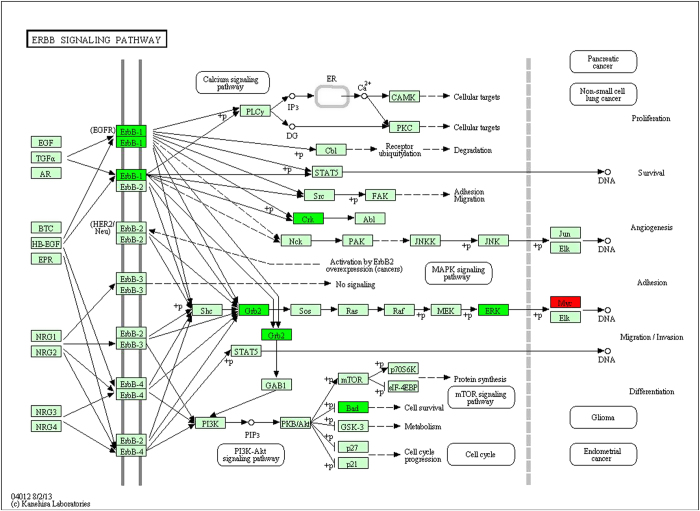
The signaling pathway of ErbB (this image was obtained by Kyoto Encyclopedia of Genes and Genomes with permission). Red represented the up-regulated genes while green represented the down-regulated genes.

108 target genes of miRNA were obtained, and there were a total of 2093 validated target genes through searching all the differential miRNA in miRWalk2.0 database. Pearson correlation tests of the 108 miRNA and their 2093 target genes were calculated respectively, and the pairs of miRNA- target genes with negative correlation value p < 0.05 were screened. There were 377 pairs which contained 202 genes and 76 miRNA. In order to study the regulation effects of the significant negative relationships, 202 genes were selected to map into the DAVID database and subjected to functional enrichment analysis. After GO enrichment and KEGG pathway analysis for the genes, we found the biological processes involved mainly included the positive regulation of the main macromolecule metabolic process, positive regulation of biosynthetic process, positive regulation of macromolecule biosynthetic process, positive regulation of gene expression and so on, among which 52 genes were enriched in 20 KEGG related pathway, and 36 genes were enriched in 13 tumor-associated pathways, including pathways in cancer, prostate cancer, chronic myeloid leukemia and CRC. In addition, 30 genes were enriched in 5 signaling pathways, including MAPK signaling pathway, ErbB signaling pathway and the like; wherein the expression of critical protein ErbB1 in ErbB signaling pathway was significantly down-regulated (Fig. 4), while 23 genes were enriched in focal adhesion and cell cycle pathway and the expression of key protein CDK2 in cell cycle pathway was up-regulated (Fig. 4).

### Screening of disease related miRNAs

**Figure 5:**
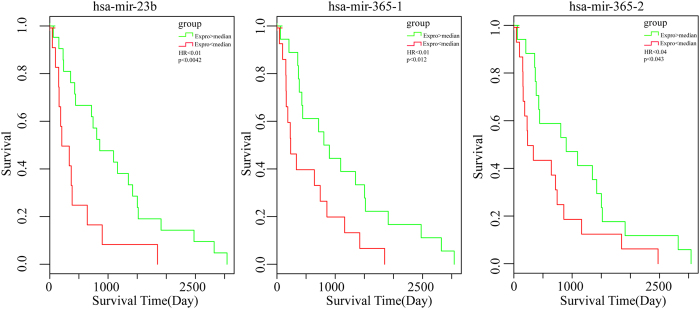
Survival analysis of 3 miRNAs. The abscissa axis showed the survival time, while the longitudinal axis showed the survival rates; the green curve in the figure indicated that Expro   > median, while the red curve indicated that Expro < median.

**Figure 6:**
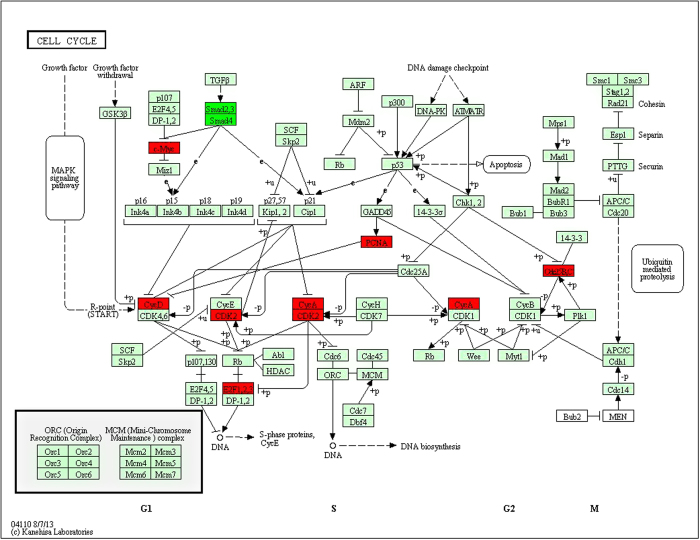
The signaling pathway of cell cycle (this image was obtained by Kyoto Encyclopedia of Genes and Genomes with permission). Red represented the up-regulated genes while green represented the down-regulated genes.

**Figure 7:**
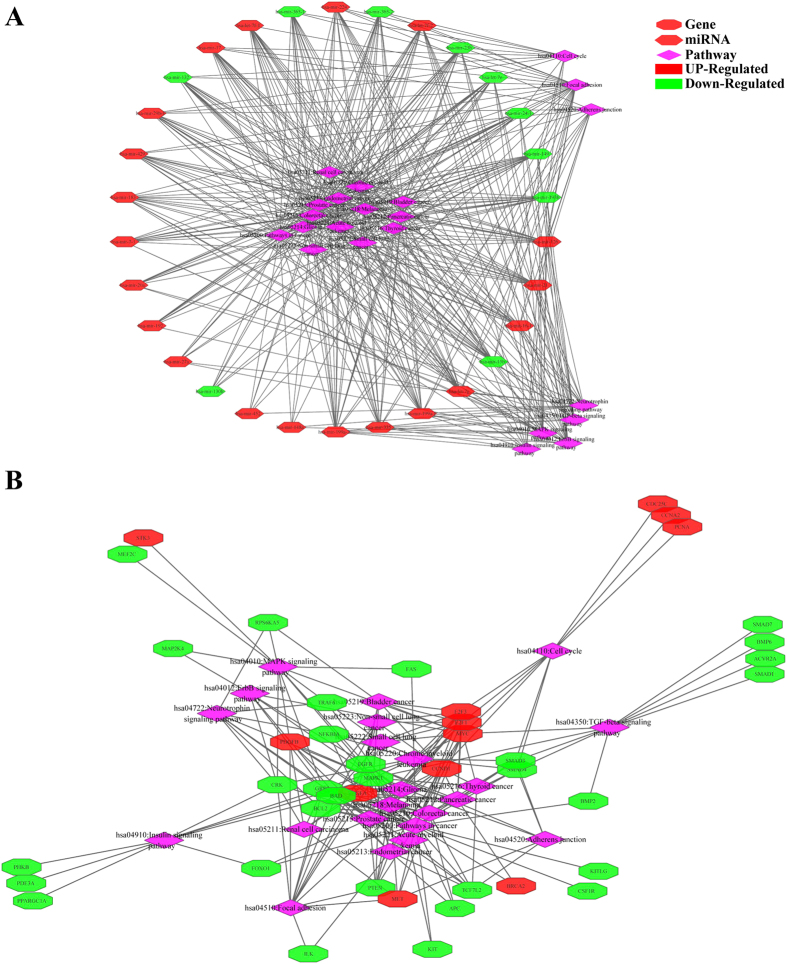
Regulation networks between miRNA and genes and pathway. (**A**) regulation networks of the miRNA and pathway; (**B**) regulation networks of the genes and pathway. Red indicated up-regulation, green indicated down- regulation.

**Figure 8:**
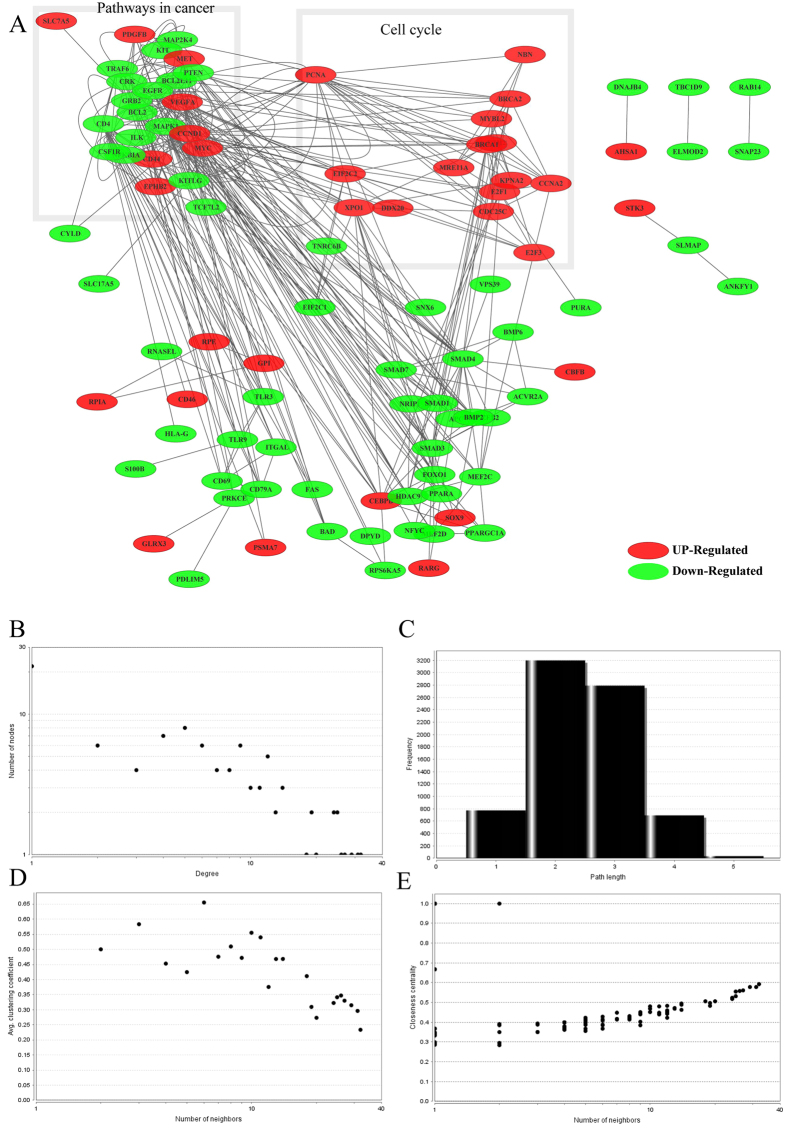
(**A**) PPI networks of disease related genes, red represented the up-regulated genes while green represented the down-regulated genes, each of side represented PPI correlations; (**B**) distribution of the degrees; (**C**) distribution of the shortest path; (**D**) average aggregation coefficient; (**E**) the proximity to the center.

With the aim of further elucidating the roles of miRNA-mRNA relationships, the miRNA-mRNA pairs where all the genes enriched in colorectal pathway were selected; then a total of 30 corresponding miRNAs were screened, finally 137 genes associated with these miRNAs were selected, which constituted 231 pairs of miRNA-mRNA with miRNA. The selected 30 miRNAs were all subjected to do survival analysis using Kaplan-Meier method, among which hsa-mir-23b, hsa-mir-365-1 and hsa-mir-365-2 showed significant influence on prognosis, as shown in Fig. 5. All these 3 miRNAs were downregulated in cancer samples, while the one with relatively high expression in the downregulated samples would have a bad prognosis. Then 13 genes in the CRC-related miRNA-mRNA pairs were subjected to do enrichment analysis, and the results showed that the main biological processes involved in included promoting the regulation of metabolic processes of large molecules, promoting the regulation of cell bio-synthesis, promoting the regulation of bio-synthesis, promoting the regulation of gene expression and so on. 42 genes were included in the KEGG pathway, among which 32 genes participated in cancer pathways, while 26 genes were associated with 5 signaling pathway and 21 genes were involved in focal adhesion, cell cycle, adhesive connection, which contained all the pathways that the original 202 genes were enriched in, indicating the selected 137 genes could represent the 202 genes with regulation procures shown in Fig. 6. Then the 137 genes were mapped to the String database and used to construct a PPI interaction network by STRING. A total of 387 pairs of protein interactions with reliability scores greater than 0.4 were selected, among which 96 nodes, accounting for 70.1% of all disease-related genes. As demonstrated in Fig. 7, the protein interaction network of these genes presented a highly aggregated state. High aggregation is an essential characteristic of biological networks. As could be seen from Fig. 7, the majority of genes in a strong interaction with each other were significantly down-regulated. And they formed two clusters, one was associated with cancer, the majority of them were down-regulated; while the other was associated with cell cycle, and most of them were up-regulated. Furthermore we calculated the topology parameters of the network, as shown in Fig. 8A–E. From A we could see the distribution of network node degrees followed a pattern of power law network; from the shortest path in B, the average aggregation coefficient in C and proximity to the center in D, we could also see that they meet the characteristics of small-world networks.

### Construction of regulation networks of miRNA-mRNA

**Figure 9:**
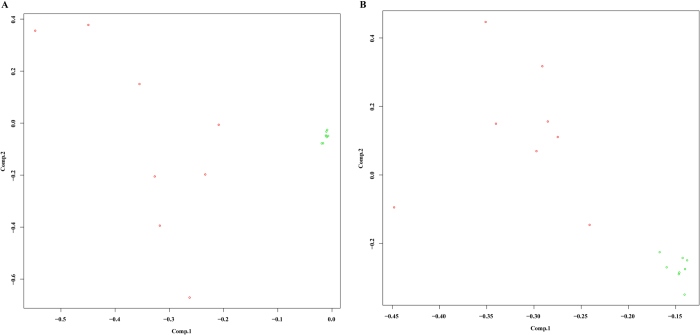
(**A**) principal component analysis of the miRNA in disease-related miRNA-mRNA pairs; (**B**) principal component analysis of the mRNA in disease-related miRNA-mRNA pairs. Green points and red points represented the randomly selected 8 normal samples and 8 disease samples respectively. The abscissa axis and longitudinal axis indicated the scores of the first principal component and the second principal component of every sample respectively.

**Figure 10:**
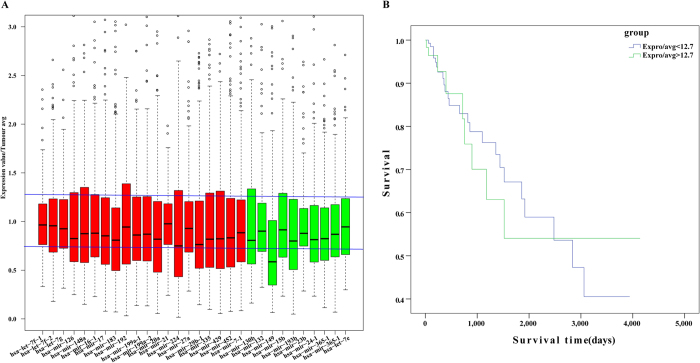
(**A**) the expression changes of various miRNA in 245 of cancer samples. Red represented the up-regulated miRNA, green represented the down-regulated miRNA. (**B**) the influence of miRNA expression on the survival rates of colorectal adenocarcinoma patients.

**Figure 11:**
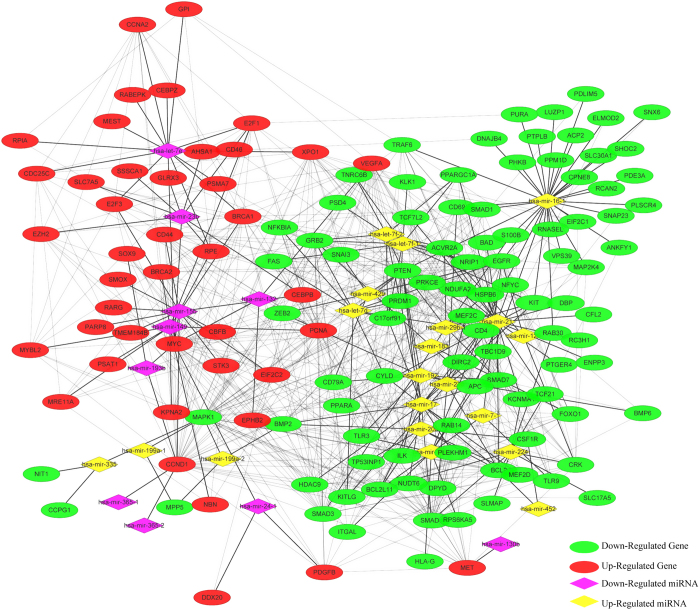
Regulation networks of miRNA-mRNA. The rhombus in yellow showed the up-regulated miRNA, the rhombus in purple showed the down-regulated miRNA, the oval in green showed the down-regulated genes and the oval in red showed the up-regulated genes.

Eight disease and also eight normal samples were randomly selected to do principal component analysis in miRNA and genes of disease-related miRNA-mRNA so as to distinguish between normal and disease samples with results shown in Fig. 9. In order to further investigate the role of miRNA in miRNA-mRNA pairs, we chose 30 miRNAs to calculate their changes in all disease samples, as shown in Fig. 10A. The majority changes of miRNAs were less than 0.25, the miRNA hsa-mir-149 with change exceeding 0.25 was selected to execute survival analysis, and observe the effect of its expression in different samples on the prognosis. The survival and treatment information of the corresponding samples in TCGA were downloaded respectively, and they were divided into two groups based on expression level of hsa-mir-149, one with high expression within the group, while the other with low expression with the final survival curve demonstrated in Fig. 10B. From which we could see the survival rates of the samples with high expression within the group were higher than that of the ones with low expression at 500–800 days; however, at 800–2500 days the survival rates were lower than that of the ones with low expression within the group, and the survival rates were higher than that of the ones with low expression within the group again after 2800 days. Ultimately a miRNA-mRNA regulatory network was successfully constructed by the regulation interaction of miRNA-mRNA (Fig. 11).

## Discussion

In the study, a total of 372 pairs of important miRNA/mRNA were found, and the potential functional relations of which were suitable for verification by an experiment. A number of such miRNA/mRNA showed they may play critical roles during the regulation of some genes, especially those expressed in cancer. For example, miR-125b inhibits the formation of tumor vessels by suppressing the expression of VE-cadherin^9^; the miRNAmiR-34 family members are important activity mediators for the p53 gene, which plays a key role in oncogenesis. Ectopic expression of miR-34 in breast cancer cells results in decreased proliferation, invasion, and induces apoptosis. Decreased expression of miR-34 was observed in breast tumors and non-small cell lung cancer^10^. In addition, the expression level of miR-221 that correlates with the expression of p53 can be regarded as a prognostic marker; miR-141 is a new biomarker that can be used for diagnostics of CRC with distant metastasis along with the tumor specific antigen CEA^6^.

In order to study the roles of the miRNA-mRNA pairs in cancer, we selected 137 genes and found that they participated in a series of biological processes and signaling pathways including MAPK signaling pathway, ErbB signaling pathway, Cell cycle and so on. The expressions of certain proteins in the MAPK signaling pathway such as MAP2K4, MAPK1, MAP3K2 and so on were all abnormal. As we known, the MAPK signaling pathway is a highly conserved intercellular signaling system present in multicellular organisms and plays an essential role in cancer progression^11^. And a number of miRNAs have been reported to be associated with the MAPK/ERK pathway in different experimental systems and tumors, some of which have been reported to directly target components of MAPK signaling, but most appear to target the downstream players of MAPK signaling, such as proteins regulating the cell cycle and migration^12^,^13^,^14^. Furthermore, the critical protein ErbB1 in ErbB signaling pathway was down-regulated.

To further study the function of the 30 miRNAs, they were all subjected to do survival analysis and we found hsa-mir-23b, hsa-mir-365-1 and hsa-mir-365-2 showed significant influence on prognosis. miR-365 has been reported to be involved in the carcinogenesis of CRC^15^, and hsa-mir-365-2 was reported to act as one of negative regulators of BCL2 through direct binding to their respective binding sites in the 3′-UTR of the human BCL2 gene^16^. While BCL2 was identified as an oncogene that does not promote cell proliferation but the evasion of cell death^17^. And previous study had proved that overexpression of hsa-mir-365-2 not only caused an increase in apoptosis but also augmented the apoptotic effect of etoposide in breast cancer MCF7 cells, indicating that hsa-mir-365-2 had therapeutic potential.

Moreover, from the expression changes of these miRNAs among various cancer samples, we found that there was little expression change in most of the miRNAs while miRNA hsa-mir-149 changed a lot. Several lines of evidence have suggested that miR149 plays multiple roles in the cell proliferation, as well as pathogenesis of the progression of various types of malignant tumors, and infectious diseases^18^,^19^,^20^. However, the results were still in dispute^21^,^22^. The expression of MiR-149 was down-regulated in several tumors, such as NSCLC, and it acted as a tumor suppressor to inhibit the oncogenes expression. For example, the expression of miR-149 was down-regulated in glioblastoma and it could restrain the proliferation and invasion of glioma cells through hindering AKT1 signaling^20^. Moreover, loss of miR-149 resulted in oncogenes expression increase and was related with tumor stage in astrocytomas and renal cell carcinoma^23^. Elevated miR-149 also played an important role in the progress of nasopharyngeal carcinoma^24^. Nevertheless, little was known about the role of miR-149 in CRC. Our study demonstrated the influence of miR-149 on CRC, we divided miR-149 into two groups based on its expression level to observe the effect on the prognosis of CRC. The results indicated that the miR-149 at different level had certain influence on the survival rates of patients with CRC.

As nearly half of the mRNAs codes protein are subjected to the miRNA-mediated regulation, the role of miRNAs as important regulators of significant genes for cancer has been confirmed^25^. Therefore, it is of importance to understand the functions of miRNA and their regulatory networks, which may provide new insights into cancer development and find new potential biomarkers and therapeutic targets. To conclude, this study may provide useful information for understanding the miRNA changes in CRC.

## Materials and Methods

### Data

The colorectal adenocarcinoma miRNASeq and level3 RNAseq data were downloaded from TCGA database. The data platforms for miRNASeq and RNASeqV2 were BCGSC__IlluminaHiSeq_miRNASeq and UNC__IlluminaHiSeq_RNASeqV2 respectively, which contained a total of 261 samples including 253 colon adenocarcinoma and eight normal tissue samples, and they were divided into two groups. The data collection and this study were conducted in compliance with all applicable laws, regulations and policies for the protection of human subjects, and any necessary approvals, authorizations, human subject assurances, informed consent documents^26^. The normalized level3 data were selected to remove the miRNA and mRNA undetected or their signals were 0. Fig. 4 of signaling pathway of ErbB and fig. 6 of signaling pathway of cell cycle were obtained by Kyoto Encyclopedia of Genes and Genomes^27^ with copyright permission.

### Screening of differential genes and miRNA

Samr^28^ packet in R software was utilized to screen the differential genes and miRNAs between normal and cancer tissues. The threshold for screening differential genes was set at delta = 1, fold change >2 and FDR <5%. In order to ensure normal and cancer tissues could be well characterized by the selected differential genes and miRNAs, we also applied limma^29^ to screen the differential genes and miRNAs, and the threshold was set at adj.P.Val = 0.05, fold change >2, finally the genes and miRNAs demonstrated to be differential by both of the algorithms were selected.

### Target genes of the differential miRNA

MiRWalk2.0^30^ database was used to inquiry all the target genes of the differential miRNAs and those had been validated by experiments (that was already reported before) were chosen. In addition, we took advantage of Pearson rank correlation to calculate the significant correlation between miRNAs and their target genes, and filtered out the genes with difference and significant negative correlation.

### Determination of the disease-related miRNA/mRNA

Firstly, the negatively regulated differential target genes were subjected to biological function enrichment analysis and the online analytic tool DAVID (Database for Annotation, Visualization and Integrated Discovery)^31^ was used to enrich GO function and KEGG pathways of the genes significantly up-regulated and down-regulated. GO terms and KEGG pathways with significant enrichment value FDR less than 0.05 were selected to be analyzed. Then the genes and their associated miRNAs enriched in CRC pathway were selected to constitute pairs of miRNA-mRNA seeds, and searched for the corresponding negatively regulated target genes of these miRNA in all the negatively regulated genes so as to form disease-related miRNA-mRNA pairs.

### Construction of PPI network

Firstly, In order to distinguish between cancer and normal tissues, the miRNA and mRNA in disease-related miRNA/mRNA pairs were chosen to perform principal component analysis (PCA). PCA is a mathematical algorithm (Raychaudhuri, Stuart *et al*. 2000), which can not only reduce the dimensionality of the data, but also retain most of the variables in the data set. Through identification of the principal components by PCA, one direction can be found, and the value of the data distributed along this direction were the maximum, which reduced the data dimension. Furthermore, just a number of variables rather than thousands of variables can be enough to classify the samples by principal component analysis. Then the disease-related miRNAs were selected and their expression changes among various cancer samples were calculated, the miRNA showing the greatest change was picked up to do survival analysis so as to screen the differential disease-related miRNA among cancers. Finally, STRING^32^ online database was applied to obtain the protein-protein interaction relationships corresponding to the genes of disease-related miRNA/mRNA, and the relationships with score coefficient greater than 0.4 were screened to build the PPI network.

### Network analysis

The regulation relationships among various genes were analyzed through calculating the topological properties of the network such as distribution of network node degree, distribution of the shortest path, the average clustering coefficient and proximity to the center and so on, moreover the related genes were subjected to depth analysis. The pairs of disease-related miRNA/mRNA were used to construct molecular regulation networks.

## Additional Information

**How to cite this article**: Zhou, X. *et al.* Identifying miRNA/mRNA negative regulation pairs in colorectal cancer. *Sci. Rep.* **5**, 12995; doi: 10.1038/srep12995 (2015).

## Figures and Tables

**Table 1:** The top ten most differential genes and miRNAs.

**Type**	**Gene ID**	**Expression change**
Gene	ZNF821|55565	Down
Gene	ZNF837|116412	Down
Gene	ZNF844|284391	Down
Gene	ZNF91|7644	Down
Gene	ZNRF1|84937	Down
Gene	ZNRF2|223082	Down
Gene	ZSWIM5|57643	Down
Gene	ZSWIM6|57688	Down
Gene	ZSWIM7|125150	Down
Gene	ZYG11B|79699	Down
Gene	? |10357	UP
Gene	? |155060	UP
Gene	AAAS|8086	UP
Gene	AADAT|51166	UP
Gene	AARS|16	UP
Gene	AARSD1|80755	UP
Gene	AASDHPPT|60496	UP
Gene	ABCC1|4363	UP
Gene	ABCC10|89845	UP
Gene	ABCE1|6059	UP
miRNA	hsa-mir-574	Down
miRNA	hsa-mir-589	Down
miRNA	hsa-mir-625	Down
miRNA	hsa-mir-652	Down
miRNA	hsa-mir-664	Down
miRNA	hsa-mir-671	Down
miRNA	hsa-mir-744	Down
miRNA	hsa-mir-766	Down
miRNA	hsa-mir-874	Down
miRNA	hsa-mir-92b	Down
miRNA	hsa-let-7f-1	UP
miRNA	hsa-let-7f-2	UP
miRNA	hsa-let-7g	UP
miRNA	hsa-mir-101-1	UP
miRNA	hsa-mir-103-1	UP
miRNA	hsa-mir-106a	UP
miRNA	hsa-mir-107	UP
miRNA	hsa-mir-10a	UP
miRNA	hsa-mir-10b	UP
miRNA	hsa-mir-126	UP
